# Genome-wide association analysis of fleece traits in Northwest Xizang white cashmere goat

**DOI:** 10.3389/fvets.2024.1409084

**Published:** 2024-05-30

**Authors:** Xiaotian Lu, Langda Suo, Xiaochun Yan, Wenze Li, Yixin Su, Bohan Zhou, Can Liu, Lepu Yang, Jiayin Wang, De Ji, Renqing Cuomu, Awang Cuoji, Ba Gui, Zhiying Wang, Wei Jiang, Yujiang Wu, Rui Su

**Affiliations:** ^1^Institute of Animal Science, Xizang Academy of Agricultural and Animal Husbandry Science, Lhasa, China; ^2^College of Animal Science, Inner Mongolia Agricultural University, Hohhot, China; ^3^Key Laboratory of Animal Genetics and Breeding on Xizang Plateau, Ministry of Agriculture and Rural Affairs, Lhasa, China; ^4^Sino-Arabian Joint Laboratory of Sheep and Goat Germplasm Innovation, Hohhot, China

**Keywords:** Northwest Xizang white cashmere goat, genome-wide association analysis, cashmere trait, GGP_Goat_70K SNP chip, linear mixed model, Sanger sequencing

## Abstract

Northwest Xizang White Cashmere Goat (NXWCG) is the first new breed of cashmere goat in the Xizang Autonomous Region. It has significant characteristics of extremely high fineness, gloss, and softness. Genome-wide association analysis is an effective biological method used to measure the consistency and correlation of genotype changes between two molecular markers in the genome. In addition, it can screen out the key genes affecting the complex traits of biological individuals. The aim of this study was to analyze the genetic mechanism of cashmere trait variation in NXWCG and to discover SNP locus and key genes closely related to traits such as superfine cashmere. Additionally, the key genes near the obtained significant SNPs were analyzed by gene function annotation and biological function mining. In this study, the phenotype data of the four traits (cashmere length, fiber length, cashmere diameter, and cashmere production) were collected. GGP_Goat_70K SNP chip was used for genotyping the ear tissue DNA of the experimental group. Subsequently, the association of phenotype data and genotype data was performed using Gemma-0.98.1 software. A linear mixed model was used for the association study. The results showed that four fleece traits were associated with 18 significant SNPs at the genome level and 232 SNPs at the chromosome level, through gene annotated from *Capra hircus* genome using assembly ARS1. A total of 107 candidate genes related to fleece traits were obtained. Combined with Gene Ontology and Kyoto Encyclopedia of Genes and Genomes enrichment analysis, we can find that *CLNS1A*, *CCSER1*, *RPS6KC1*, *PRLR*, *KCNRG*, *KCNK9,* and *CLYBL* can be used as important candidate genes for fleece traits of NXWCG. We used Sanger sequencing and suitability chi-square test to further verify the significant loci and candidate genes screened by GWAS, and the results show that the base mutations loci on the five candidate genes, *CCSER1* (snp12579, 34,449,796, A → G), *RPS6KC1* (snp41503, 69,173,527, A → G), *KCNRG* (snp41082, 67,134,820, G → A), *KCNK9* (14:78472665, 78,472,665, G → A), and *CLYBL* (12: 9705753, 9,705,753, C → T), significantly affect the fleece traits of NXWCG. The results provide a valuable basis for future research and contribute to a better understanding of the genetic structure variation of the goat.

## 1 Introduction

Cashmere goats, as a part of global biodiversity, have experienced a long process of natural selection and artificial breeding. Nowadays, they have become one of the breeds with the highest cashmere production and excellent cashmere quality in the world. The most important economic value of the cashmere goats derives from its cashmere. Cashmere has the characteristics of fine and soft, white color as snow, bright color, and delicate and smooth feel. Textiles made of cashmere are unique in lightness, comfort, warmth, and elegance. NXWCG is the first new breed of Cashmere Goat cultivation in Xizang Autonomous. Due to the particularity of its living environment, the NXWCG has the characteristics of hypoxia tolerance and high cold tolerance. NXWCG has the most pure blood, the finest cashmere, the largest hair follicle density, and the uniform thickness of a single fiber. Based on its significant characteristics of extremely high fineness, gloss, and softness, we selected this breed of cashmere goat as the experimental object for related genetic research.

The fleece traits are mostly one of the quantitative traits in the cashmere goats. Quantitative traits refer to traits that are controlled by a small number of major genes and gene networks, a large number of minor genes, and environmental modifications; there are interactions between genes and the environment, and gene expression is affected by other genes, biological small molecules, and gene methylation. The fleece quality traits are traits with continuous variation or non-Mendel intermittent variation (1, 2). In brief, the variation of cashmere quality is continuous, and the variation is easily affected by environmental conditions. The earliest genetic improvement method of quantitative traits is to select individual phenotype traits, including individual selection method, family selection method, and mixed selection method. With the introduction of the concept of quantitative genetics in the 1920s, quantitative genetics has undergone continuous development. It has achieved remarkable results in analyzing the genetic mechanism and biological evolution of quantitative traits (3, 4).

Molecular genetics is a branch of genetics that studies the mechanism of biological inheritance and variation at the molecular level. The main research contents of molecular genetics include the nature of the gene, the function of the gene, the change of the gene, and the structure and function of the protein. In the breeding of cashmere goats, the method of combining phenotype- and genotype-assisted selection is gradually improved, which provides theoretical support for improving the accuracy and efficiency of selection and shortening the generation interval. Duan et al. found that cashmere goats with curly fiber hair can produce higher production and finer fiber hair than straight cashmere goats by studying the cashmere characteristics of Yanshan cashmere goats, and this difference in fiber characteristics can be related to the mutation of *KRT1* and *KRT6A* genes (5). Li et al. used the iRTAQ-based method and identified that different protein abundances are associated with different fiber characteristics. Different protein abundances mainly refer to keratin or keratin-associated proteins (*KRTAP11-1*, *KRT6A*, and *KRT38*). Transcriptome sequencing confirmed that the *DSC2* gene was significantly associated with the fleece phenotype of goat (6). Purvis and Franklin found that keratin intermediate protein and keratin-associated protein play an important role in determining different fleece quality and production traits (7). These studies suggest that hair follicle and hair fiber-related genes are involved in skin structure and fleece development, and the diversity of these genes may have an impact on the structure and traits of fleece. DNA molecular marker is the genome roadmap. Molecular markers are widely used in genetic map construction, population genetic diversity analysis, marker-assisted selection breeding in animal breeding, gene mapping, and cloning. SNP marker is a single nucleotide polymorphism molecular marker based on PCR and sequencing technology. The mutation rate of SNP in the genome is very low, which can effectively distinguish different alleles and cover the whole genome with high density, low cost, and easy automation of high-throughput analysis (8, 9). Sun et al. reveal the potential genetic basis for litter size, coat color, and skin color by using genome-wide association analysis (GWAS), selection signature analysis, and ROH detection within the Youzhou dark goat population through GWAS; four SNPs were identified (10). Mukhina et al. evaluated the genetic risk of five Mongolian goat breeds (Buural, Ulgii Red, Gobi GS, Erchim, and Dorgon) using Illumina Goat SNP50 genotyping data and explored the phylogenetic relationship between these populations and other breeds (11).

Genome-wide association study (GWAS) is an analysis method based on the principle of linkage disequilibrium. By detecting the genetic variation polymorphism in the whole genome of the associated population composed of hundreds of individuals, tens of thousands or even millions of molecular markers are obtained, and then the relationship between the target traits and the molecular markers is identified, and the relationship between the genetic variation and the characteristics of the population samples is determined. GWAS is widely used in the genetic variation of non-disease quantitative trait variation such as height and weight (12–14). After several years of accumulation and summary of genomic association analysis theory, Klein et al. from Rockefeller University published a GWAS study on age-related macular degeneration in 2005 (15). The study by Klein et al. opened the prelude of GWAS in human, animal, and plant genetic research (16, 17).

In recent years, to accelerate the process of animal genetic improvement and overcome some difficulties in animal genetic breeding, genome-wide association analysis using genotype data obtained using genome resequencing and genotyping technology and animal phenotype trait data has become increasingly mature (18–20). GWAS technology is becoming more and more powerful with the development of next-generation and third-generation sequencing technology.

Sanger sequencing is the international gold standard for many gene detection technologies. This sequencing technology can well detect the accuracy of chip sequencing technology, second-generation sequencing technology, third-generation sequencing technology, and up-to-date fourth-generation sequencing technology. Takenaka quantified RNA editing efficiency by comparing base mutations in the Sanger sequencing chromatogram of RT-PCR products because its sequencing accuracy is superior to other sequencing methods (21). Studies show that Sanger sequencing can effectively and rapidly detect and quantify multiple low VAF BRAF mutations from FFPE samples (22). Danilov et al. compared Bead Chip and WGS/WES genotyping calls via Sanger sequencing, and the results showed that the average precision and accuracy of gene chip and WGS were more than 0.991 and 0.997, respectively (23). The Sanger sequencing method is widely used in the analysis of biological genetic mechanisms.

## 2 Materials and methods

### 2.1 Ethics statement

The feeding environment of this experiment is in line with the relevant standards of ordinary animal experimental facilities in the Chinese national standard “Laboratory Animal Environment and Facilities” (GB14925-2010). The feeding and experimental operation of animals meets the requirements of animal welfare. All the experimental procedures involving goats were reviewed and approved by the Experimental Animal Management Committee of Inner Mongolia Agricultural University, Hohhot, China.

### 2.2 Sample collection and obtaining phenotype data

In totally, 539 samples were collected from the NXWCG Original Breeding Farm in Ritu County, Ali Prefecture, Xizang Autonomous Region. Among them, 288 samples were used for the GWAS study and 251 samples were used for the validation test. It contains 335 breeding rams and 204 ewes. The production performance parameters, such as cashmere length and fiber length of the experimental group, were measured in the field of the Original Breeding Farm, and the cashmere and ear tissue samples of the experimental group were collected and brought back to the laboratory for further study.

Descriptive statistical analysis of phenotype data was performed using R software. The ggplot2 package of R software was used to draw the frequency distribution histogram and fitting curve of the phenotype, and the normal distribution law of each trait was judged by graphics.

### 2.3 Obtaining genotype data and quality control

The genotype data of this experiment were derived from the ear tissues of cashmere goats collected from the Original Breeding Farm of Northwest Xizang white cashmere goats. The ear tissues were collected and placed in a PE tube containing 75% anhydrous ethanol and stored in a refrigerator at −80°C. The DNA of ear tissues was extracted by phenol extraction method in the laboratory. The concentration of DNA was detected by NANODROP 2000c (Thermo, Waltham, MA, USA). The extracted genomic DNA was detected by agarose gel electrophoresis. There were clear bands with a length of more than 10 kb and no obvious degradation. The OD260/280 value of DNA samples should be between 1.7 and 2.1.

The DNA samples that met the requirements of phenotype data and DNA concentration were screened and placed in a 96-well PCR plate, and the genotype sequencing based on the goat 70 K chip was performed by Neogen Bio-Scientific Technology (Shanghai) Co., Ltd.

Plink1.90 software was used to control the quality of genotype data to eliminate unqualified samples and SNP loci. Quality control standards are as follows: (1) SNP deletion quality control: SNP genotype deletions greater than 10% (−geno0.1) were removed from the analysis; (2) Sample missing quality control: samples with a call rate lower than 90% (−mind0.1) were excluded from the analysis; (3) MAF deletion quality control: loci with a minimum allele frequency of less than 1% (−maf0.01) were excluded from the analysis; (4) Hardy–Weinberg equilibrium quality control: sites that did not meet the Hardy–Weinberg equilibrium test with a *p*-value of less than 10^−6^ were excluded from the analysis.

To determine whether the SNPs after quality control have sufficient coverage in the whole genome, Haploview4.1 software was used to calculate the LD coefficient between SNP markers and the distance between markers. Excel software is used to calculate the average value of the LD coefficient corresponding to each distance; finally, the average value is used to draw the LD attenuation diagram.

### 2.4 GWAS

In this study, the mixed linear model of GEMMA-0.98 software (Genome-wide Efficient Mixed Model Association algorithm) was used to perform genome-wide association analysis on the phenotype and genotype values of four fleece traits in the individuals after genotype quality control (24). Individual age is added to the mixed linear model as a fixed effect. In this study, y=Xα+Zβ+Wμ+e statistical analysis model was used, where y is the phenotype trait, X is the fixed effect matrix, α is the fixed effect estimation parameter, Z is the single nucleotide polymorphism matrix, β is the single nucleotide polymorphism effect, W is the random effect matrix, μ is the predicted random individual, e is the random error, and the distribution is $e\sim N\;\left(\;0,\sigma_{e}^{2}\;\right)$ .

Through the principal component analysis of the population genotype data, the principal component of the population genetic variation is calculated, that is, the genetic variation of the population is compressed into several smaller dimensions, and then it is defined as the eigenvector of the inter-individual covariance matrix. The principal component is added as a covariate to the subsequent GWAS analysis model to correct the potential population stratification of the associated population. It is necessary to consider the interference factors caused by population stratification, and it is necessary to perform population stratification detection and parameter correction on the experiment population. In this study, the principal component analysis of the experiment population was carried out using Plink software. The first two principal components were extracted by R software, and the scatter plot of principal component analysis was drawn to observe whether there were differences in the genetic background of the population. Individual age and the first three principal components were added to the mixed linear model as covariates to correct the phenotype data.

The genome-wide association analysis of four phenotype traits (cashmere length, cashmere diameter, fiber length, and cashmere production of NXWCG) was carried out by Gemma-0.98.1 software under the Linux system. First, the population genome kinship matrix is calculated, and then the covariate and Kinship matrix are added to the model for data processing.

The output result files by Gemma software were sorted out, and the parameters used to draw the Q-Q diagram and Manhattan diagram were screened. R software was used to draw visual graphics of four trait variation sites. In order to eliminate the false negative caused by too strict Bonferroni correction, and to explore more significant SNPs loci and candidate genes affecting the fleece traits of NXWCG, combined with the results of association analysis in this study, when the potentially significant level threshold was 1 × 10^−3^, that correspond to 3 in -log_10_ (*p*-value) scale, it was determined that it was significantly associated with the traits studied (25, 26).

### 2.5 Gene annotation and bioinformatics analysis

The goat reference genome data (Genome assembly ARS1.2) was downloaded from the website of the National Center for Biotechnology Information^1^; the candidate genes within 50 kb upstream and downstream of the significant sites were annotated by bedtools software.

GO function annotation and KEGG signaling pathway analysis were performed using DAVID6.8^2^ database. *p* < 0.05 is statistically significant in the results of enrichment analysis. GO and KEGG histograms and bubble plots were plotted using the ggplot2 package of R software.

### 2.6 Validation analysis of GWAS results

In this study, Sanger sequencing method was used to verify the results of GWAS analysis. The ear tissue DNA samples used for Sanger sequencing were all from NXWCG Original Breeding Farm. The DNA samples were different from the GWAS study. The ear tissue DNA of 96 individuals was screened for each fleece trait.

In this study, PCR primers were designed based on the 300 bp upstream and downstream chromosome fragments of 8 significant SNP loci obtained by GWAS (Table 1). Primer design and preparation were carried out by Sangon Biotech (Shanghai) Co., Ltd. (Table 2). PCR amplification was performed using the T100 Thermal Cycler (Bio-Rad), the Composition of conventional PCR reaction solution, and the PCR reaction conditions as follows (Tables 3, 4).

**Table 1:** SNP locus verification table.

Traits	Chr	SNP loci	SNP	*p*-value	Gene
CL	6	34,449,796	snp12579	1.16E-04	*CCSER1*
CD	16	69,173,527	snp41503	4.50E-06	*RPS6KC1*
FL	12	67,134,820	snp41082	2.84E-05	KCNRG
CP	14	78,472,665	14:78472665	3.83E-05	KCNK9
	12	9,705,753	12:9705753	4.99E-05	CLYBL

CL, cashmere length; CD, cashmere diameter; FL, fiber length; CP, cashmere production.

**Table 2:** The primer sequence information of PCR amplification.

Gene	Primer Sequence		Product size/bp
*CCSER1*	F	5’ GTGTTATCCCTAGAGAGCCTGTTTC 3’	247
	R	5’ AGCATGAACCTCAAAGACTGGAAG 3’	
*RPS6KC1*	F	5’ GCATCCACAGTTCCAGCAGAAG 3’	263
	R	5’ CAGAAGCCCAGCACTCGTTTG 3’	
*KCNRG*	F	5’ CTGTGGGAGATAAAGATAGGGCATC 3’	283
	R	5’ AAGTGTTAGTGACTGTAAGCCAAGG 3’	
*KCNK9*	F	5’ GCCTCTCTGGAATATCCTCCTATGG 3’	214
	R	5’ CATCGCCTTCTCTGGTTTGTTGG 3’	
*CLYBL*	F	5’ AACAAGAGAAACCAGCGAAAGGAG 3	218
	R	5’ ACACGAGAGCCACTGGAATGAG 3’	

**Table 3:** Composition of the conventional PCR reaction solution.

Reagent	Amount
*TaKaRa Ex Taq*®(5 U/μl)	0.25 μL
10 × *Ex Taq Buffer* (Mg^2+^plus)(20 mM)	5 μL
dNTP Mixture (2.5 mM each)	4 μL
Template	3 μL
Primer F	3 μL
Primer R	3 μL
ddH_2_O	31.75 μL

**Table 4:** Conventional PCR reaction conditions.

Step	Temperature	Time		Cycle
Degeneration	98°C	10s	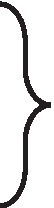	35
Annealing	58°C-62°C	30s		
Elongation	72°C	1 min		
Elongation	72°C	5 min		1
Preservation	4°C	∞		1

The unpurified samples, after PCR amplification, were sent to Sangon Biotech (Shanghai) Co., Ltd. for capillary electrophoresis sequencing (CE). The sequencing was performed using the 3730xl DNA Analyzer (Thermo Fisher). Finally, the peak color map in ab1 format and the sequence file in seq format were generated for genotyping statistics. The chisq.test function of R software was used to test the fitness of candidate genes, and the chi-square value and *p*-value value of each candidate gene were obtained.

## 3 Results

### 3.1 Basic statistical analysis of phenotype data

**Figure 1:**
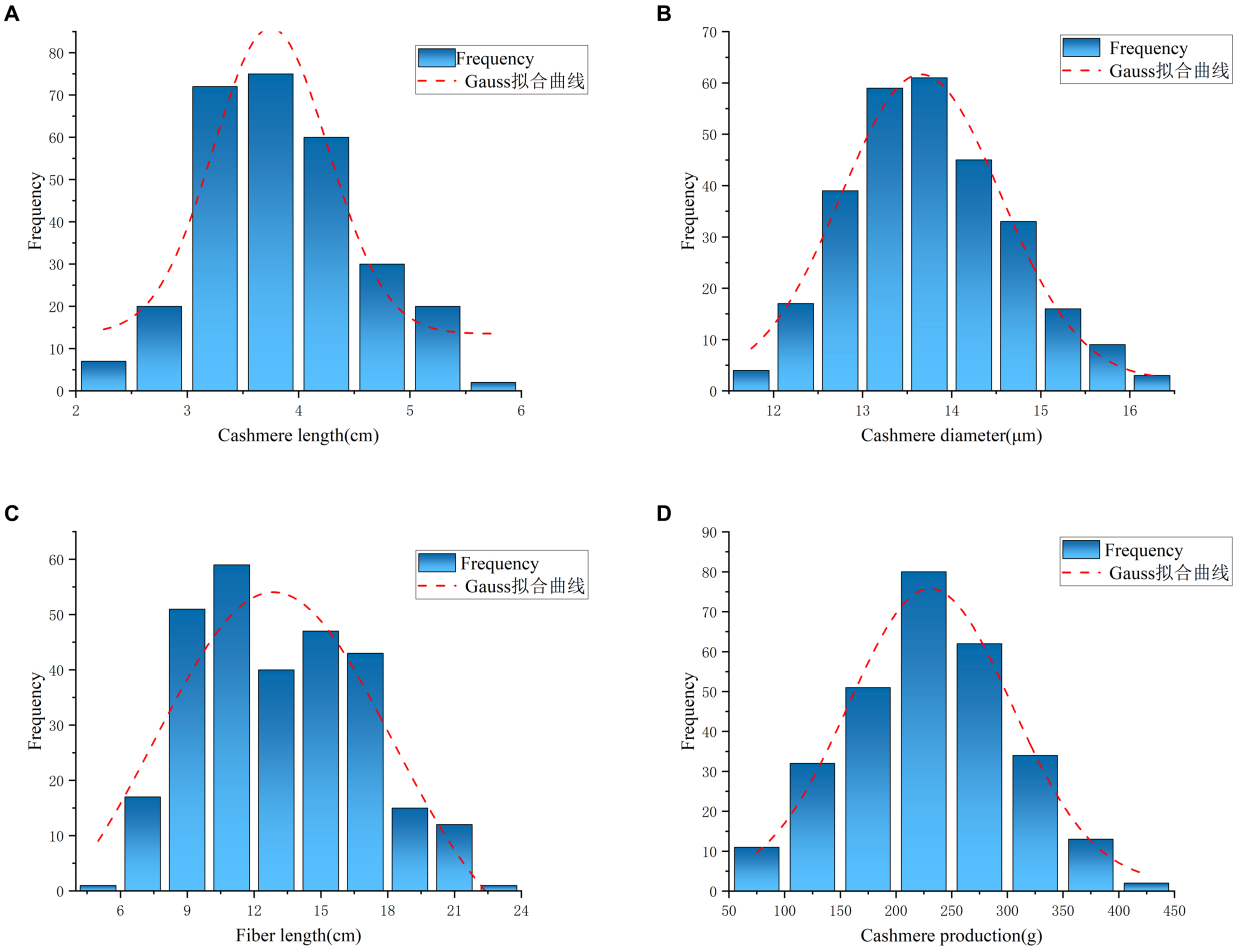
Frequency distribution histogram for fleece traits. **(A)** Indicate the frequency distribution and fitting curve of Cashmere length trait. **(B)** Indicate the frequency distribution and fitting curve of Cashmere diameter trait. **(C)** Indicate the frequency distribution and fitting curve of Fiber length trait. **(D)** Indicate the frequency distribution and fitting curve of Cashmere production trait.

In this study, the fleece traits of 288 NXWCG for GWAS were measured, including cashmere length, cashmere diameter, fiber length, and cashmere production. The phenotype data were sorted out, and descriptive statistical analysis was performed on the four traits (Table 5). According to the frequency distribution histogram and fitting curve of each phenotype (Figure 1), it was judged that each trait tended to normal distribution and could be used for subsequent genome-wide association analysis.

**Table 5:** Descriptive statistics of phenotype values of cashmere traits.

Traits	Number	Mean	SD	Max	Min	CV
CL (cm)	288	3.79	0.73	5.90	2.10	19.26%
CD (μm)	288	13.75	0.91	16.41	11.68	6.62%
FL (cm)	288	12.71	3.64	22.00	5.00	28.63%
CP (g)	288	229.8	72.28	426.9	64.0	31.45%

CL, cashmere length; CD, cashmere diameter; FL, fiber length; CP, cashmere production.

### 3.2 Quality control of genotype data and analysis

**Figure 2:**
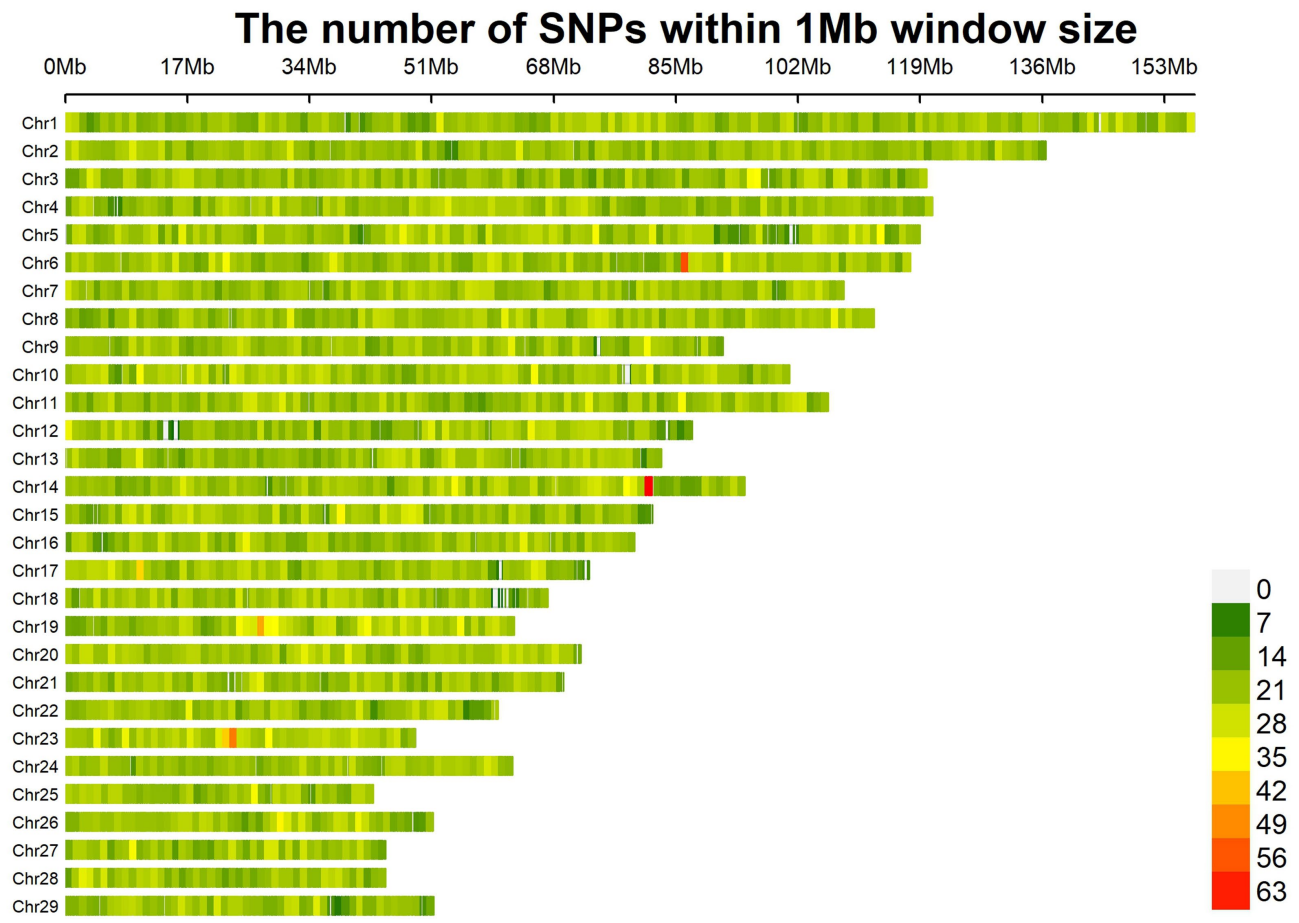
Distribution of SNPs in chromosome.

**Figure 3:**
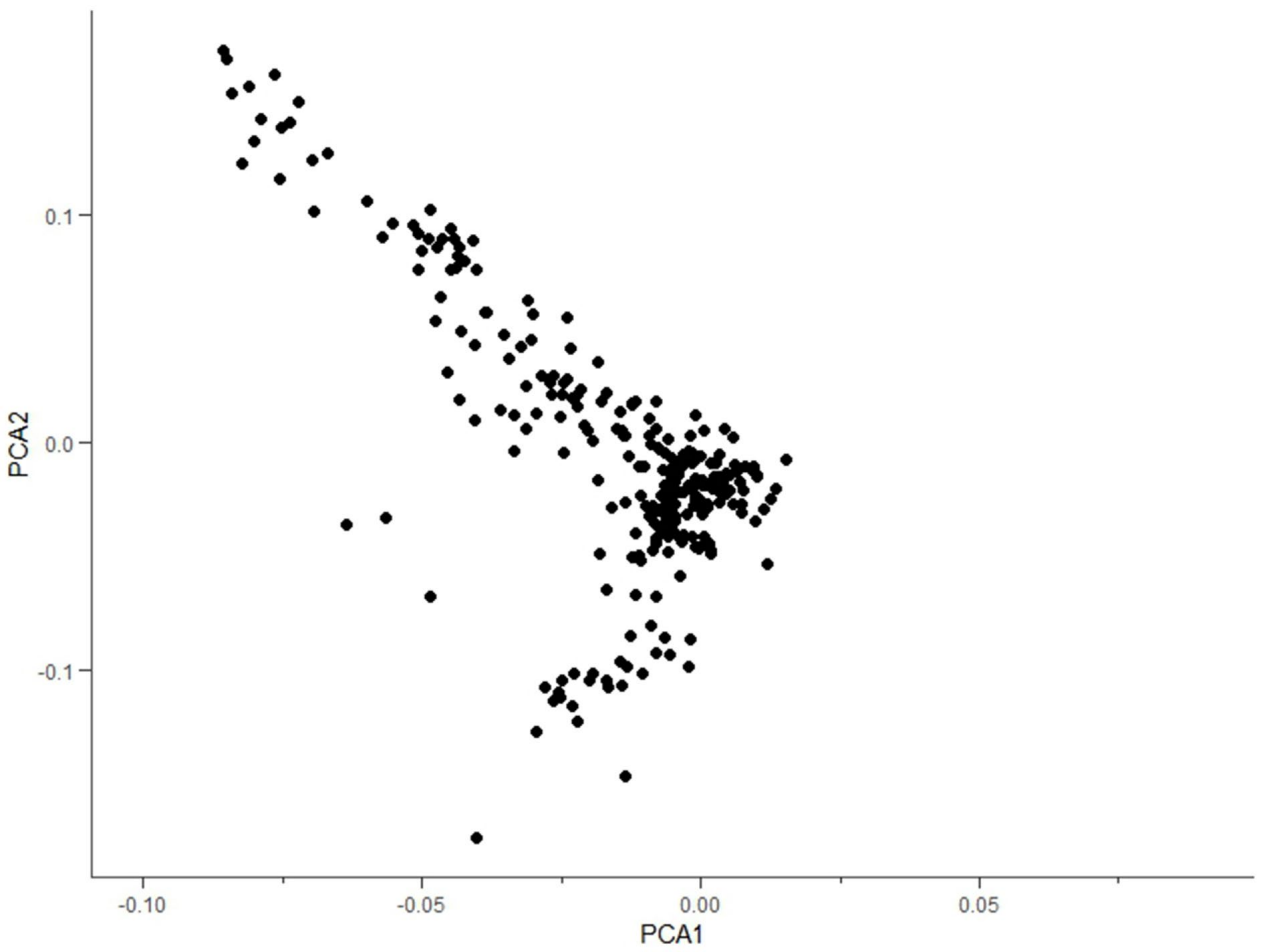
Principal component analysis.

A total of 67,088 SNP loci were obtained after genotyping 288 individual ear tissue DNA. Through genotype data quality control, 1,276 SNPs were excluded from the quality control of single nucleotide site deletion, two individuals were excluded from the sample missing quality control, 10,242 SNPs were excluded from MAF quality control, 1,890 SNPs were excluded from Hardy–Weinberg equilibrium, and 523 SNPs on sex chromosomes were removed. A total of 53,157 SNPs were obtained after quality control of raw data, which were uniformly distributed on 29 pairs of autosomes of goats (Figure 2). To reduce the false positive results of genome-wide association analysis, it is necessary to analyze the population structure of 286 individuals and add the first three principal components of the population as covariates to the GWAS model. The principal components analysis results showed that there was no obvious stratification in the group, indicating that there was almost no alien lineage between the samples (Figure 3).

**Figure 4:**
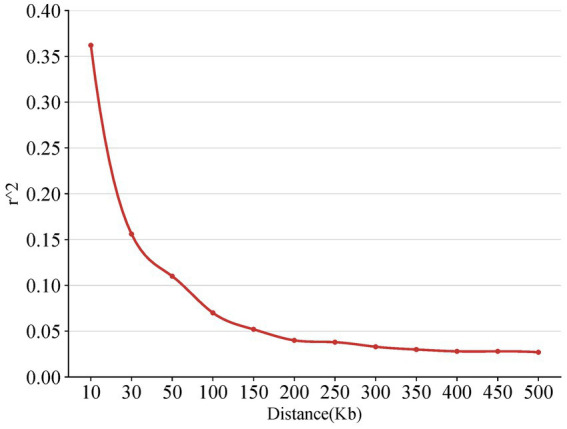
The diagram of the linkage disequilibrium.

The LD decay map is a two-dimensional curve to show the process that the average LD coefficient between SNP markers on the chromosome of the genome decreases linearly with the increase of the distance between markers. The linkage disequilibrium also measures the genotype synchronization and correlation index of the two molecular markers. In this study, the LD decay map showed that the LD correlation coefficient (*r*^2^) decreased gradually after the physical distance reached 200 kb, indicating that the closer the distance between the two SNP loci on the chromosome, the stronger the correlation and the greater the LD coefficient (Figure 4).

### 3.3 GWAS

**Figure 5:**
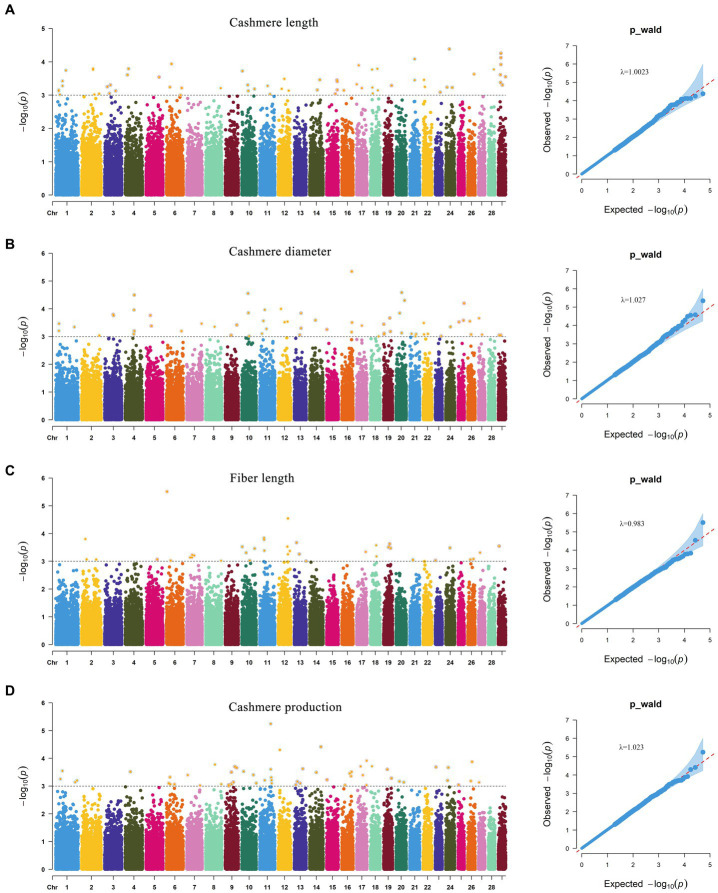
Manhattan and Q-Q plot of fleece trait. **(A)** Indicate the Manhattan and Q-Q plot of Cashmere length trait. **(B)** Indicate the Manhattan and Q-Q plot of Cashmere diameter trait. **(C)** Indicate the Manhattan and Q-Q plot of Fiber length trait. **(D)** Indicate the Manhattan and Q-Q plot of Cashmere production trait.

GWAS results of cashmere length showed that 77 SNPs were significant at the potentially significant level (Figure 5A). The calculation results of the expansion factor of the Q-Q diagram show that there was no genome expansion, indicating that the model used had a good fitting degree and was suitable for this trait. Through gene annotation, 18 candidate genes related to cashmere length traits were obtained. For the cashmere diameter trait, 71 SNPs were significant at the potentially significant level. The gene expansion factor was *λ* = 1.027 (Figure 5B); through gene annotation, 32 candidate genes related to the cashmere diameter trait were obtained. For the fiber length trait, 42 SNPs were significant at the potentially significant level. The gene expansion factor was *λ* = 0.983 (Figure 5C); through gene annotation, 18 candidate genes related to fiber length trait were obtained. For the cashmere production trait, 60 SNPs were significant at the potentially significant level. The gene expansion factor was *λ* = 1.023 (Figure 5D); through gene annotation, 25 candidate genes related to cashmere production trait were obtained (Table 6). Table 6 lists some of the more significant loci and annotated genes in the GWAS results of four fleece traits, respectively.

**Table 6:** Gene list of GWAS candidate genes of fleece traits.

Traits	SNP	Chr	Position (bp)	Area	*p-*value	Gene
CL	NC_030836.1_17,791,205	29	17,791,205	intron	7.48E-05	*CLNS1A*
	snp12579	6	34,449,796	intron	1.16E-04	*CCSER1*
	29:17721108	29	17,721,108	intron	1.20E-04	*RSF1*
	snp57618	2	79,623,520	intron	1.60E-04	*LRP1B*
	snp14053	4	22,588,864	intron	1.61E-04	*EXOC4*
	snp33206	18	47,647,221	cds	1.62E-04	*CAPNS1*
CD	snp41503	16	69,173,527	intron	4.50E-06	*RPS6KC1*
	snp4438	20	38,993,710	intron	2.59E-05	*PRLR*
	10:42743991	10	42,743,991	intron	2.77E-05	*ATP8B4*
	snp31138	4	62,616,471	intron	3.18E-05	*IMMP2L*
	12:17532273	12	17,532,273	intron	1.01E-04	*GPC6*
	10:45283375	10	45,283,375	intron	1.40E-04	*MYO5A*
FL	snp41082	12	67,134,820	intron	2.84E-05	*KCNRG*
	snp41082	12	67,134,820	intron	2.84E-05	*LOC102188416*
	NC_030818.1_29,807,979	11	29,807,979	intron	1.43E-04	*FBXO11*
	snp56066	13	15,313,527	intron	2.12E-04	*CELF2*
	19:42091591	19	42,091,591	intron	2.35E-04	*STAT3*
	18:39512710	18	39,512,710	intron	2.64E-04	*ZFHX3*
CP	14:78472665	14	78,472,665	intron	3.83E-05	*KCNK9*
	12:9705753	12	9,705,753	intron	4.99E-05	*CLYBL*
	snp52367	17	69,328,879	intron	1.21E-04	*CPE*
	NC_030825.1_9,378,797	18	9,378,797	intron	1.94E-04	*LOC102181146*
	snp46857	17	27,541,955	3’-UTR	1.95E-04	*GUCY1A3*
	snp50516	9	66,936,671	intron	1.99E-04	*ADGRG6*

CL, cashmere length; CD, cashmere diameter; FL, fiber length; CP, cashmere production.

### 3.4 GO and KEGG analysis

**Figure 6:**
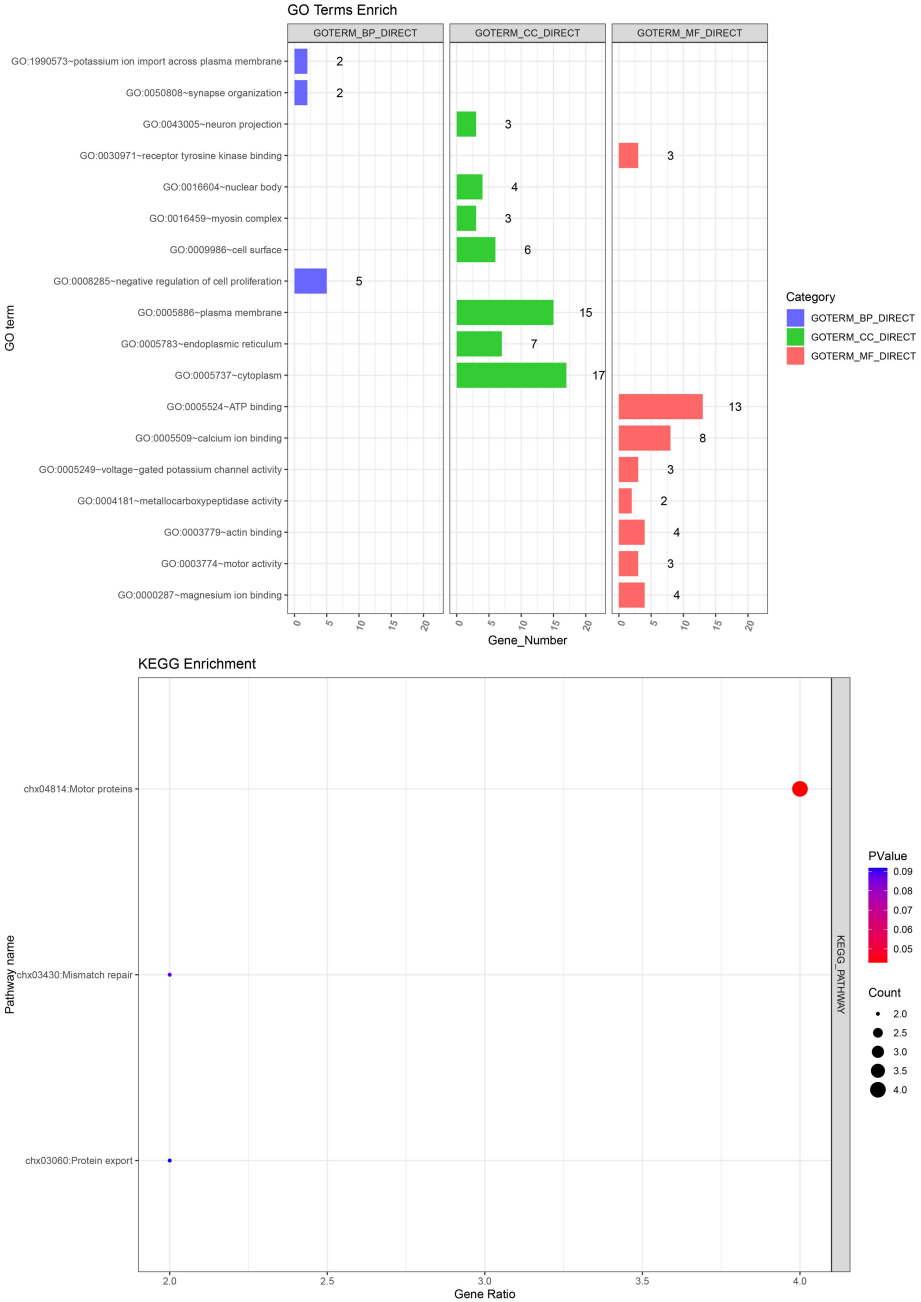
GO and KEGG enrichment analysis.

The GO and KEGG enrichment analysis of 107 candidate genes associated with fleece traits was performed by NCBI‘s DAVID 2021 Gene system (Table 7). Gene ontology was enriched to one biological process, five cellular components, and five molecular functions (*p* < 0.05). GO enrichment to the negative regulation of cell proliferation biological processes (GO: 0008285) includes *ALDH1A2*, *STAT3*, *CDH13,* and five other genes. It is enriched in cellular components such as cytoplasm (GO: 0005737), cell membrane surface (GO: 0009986), and endoplasmic reticulum (GO: 0005783), including genes such as *CREBBP*, *PRLR,* and *KCNRG*. The enriched molecular functions included ATP binding (GO: 0005524), calcium ion binding (GO: 0005509), and voltage-gated potassium channel activity (GO: 0005249), including *RPS6KC1*, *CAPNS1*, and *KCNK9* genes. KEGG was enriched to one signal pathway of motor protein (chx: 04814) (*p* < 0.05), and *MYO15A*, *KIF16B*, *MYO5C* and *MYO5A* were significantly enriched to this pathway (Figure 6).

**Table 7:** GO function and KEGG signaling pathway enrichment table.

Category	Term	Count	*p-*value	Gene
BP	negative regulation of cell proliferation	5	0.0150	*ALDH1A2, STAT3, CDH13*, etc
CC	cytoplasm	17	0.0045	*CREBBP, ZFHX3, DENND4A*, etc
CC	cell surface	6	0.0082	*ADGRG6, TNR, PRLR*, etc
CC	endoplasmic reticulum	7	0.0088	*KCNRG, HSPA13, CDS2*, etc
CC	myosin complex	3	0.0128	*MYO15A, MYO5C, MYO5A*
CC	neuron projection	3	0.0293	*GRM5, KCNQ1, CDH13*
MF	ATP binding	13	0.0101	*RPS6KC1, NARS2, MYO5C*, etc
MF	calcium ion binding	8	0.0111	*CAPNS1, PITPNM3, CDH13*, etc
MF	motor activity	3	0.0116	*MYO15A, MYO5C, MYO5A*
MF	receptor tyrosine kinase binding	3	0.0139	*PCNA, PITPNM3, PTPN14*
MF	voltage-gated potassium channel activity	3	0.0186	*KCND2, KCNK9, KCNH1*
KEGG	Motor proteins	4	0.0429	*MYO15A, KIF16B, MYO5A*

BP, Biological process; CC, Cellular component; MF, Molecular function.

### 3.5 Validation analysis of GWAS

**Figure 7:**
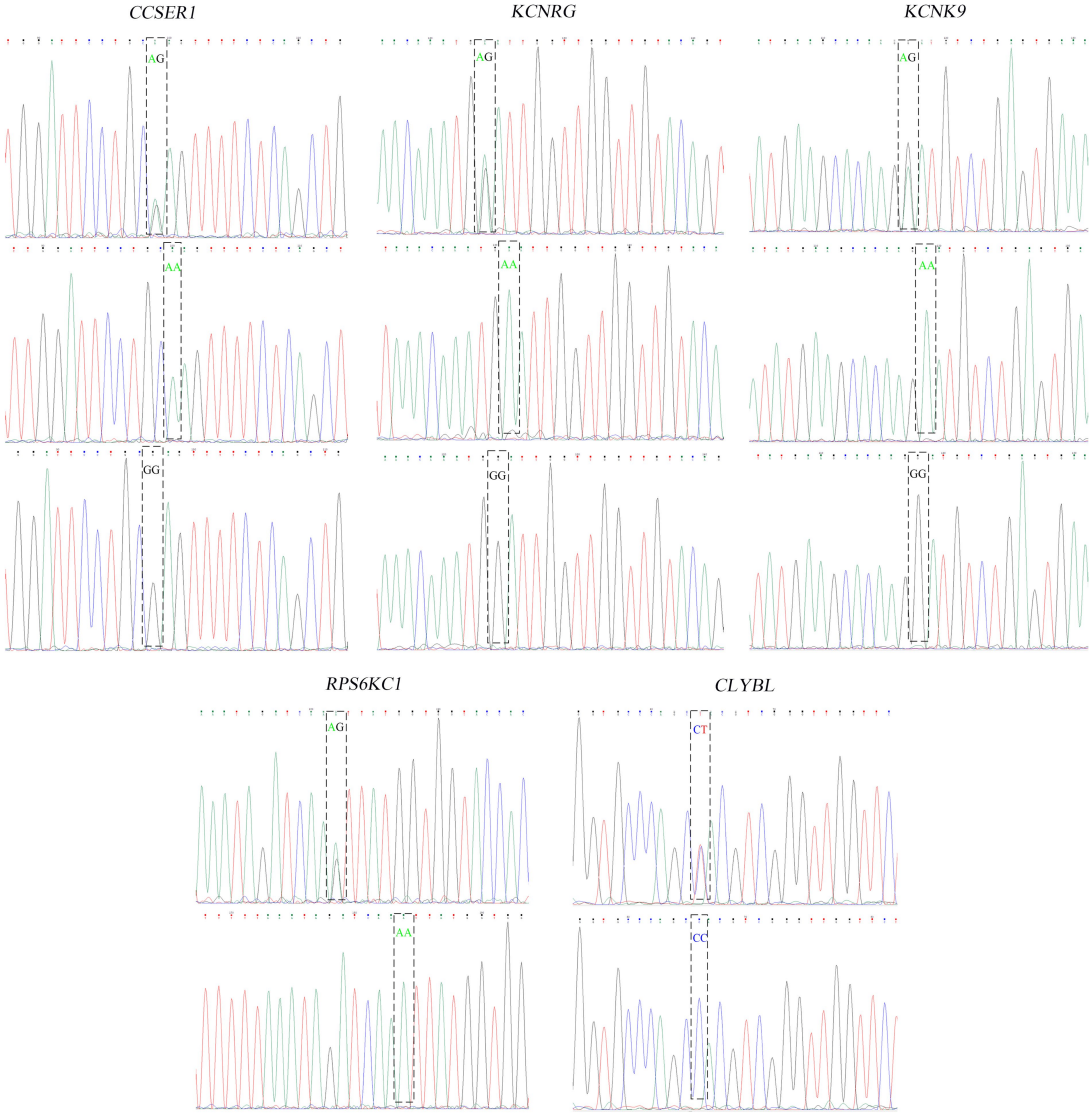
Peak color map of Sanger sequencing.

The one or two most significant loci of each trait in GWAS results were selected for the verification test. In total, 5 pairs of primers for four fleece traits of NXWCG were pre-tested. Finally, 5 pairs of primers successfully amplified the target length fragment altogether. Based on 5 pairs of primers, PCR amplification was performed on 251 individual ear tissue DNA templates of NXWCG. Sanger sequencing was performed on 251 amplified sequences. The results of Sanger sequencing were analyzed using Chromas 2.6.6 software. Finally, 5 chromosome mutation sites were found (Figure 7). The candidate genes corresponding to the five chromosome mutation sites were genotyping statistics. The chi-square test was used to analyze the correlation between significant loci and phenotype traits (Table 8). Verification analysis results show that the mutation sites of *CCSER1* (snp12579,34,449,796, A → G), *RPS6KC1* (snp41503,69,173,527, A → G), *KCNRG* (snp41082, 67,134,820, G → A), *KCNK9* (14: 78472665,78,472,665, G → A), and *CLYBL* (12: 9705753,9,705,753, C → T) were consistent with the results of GWAS analysis and highly significant (*p* < 0.01). The accuracy of genome-wide association analysis of fleece traits of NXWCG was verified theoretically.

**Table 8:** Genotyping statistics and Chi-square test table.

Traits	Genes	Genotype	Number	Mean	SD	CV	*p*-value
CL	*CCSER1*	AA	29 (60.4%)	3.77	1.22	32.36%	0.00017
		AG	13 (27.1%)	3.46	1.10	31.79%	
		GG	6 (12.5%)	3.80	1.13	29.74%	
CD	*RPS6KC1*	AA	30 (83.3%)	13.76	1.18	8.58%	0.00006
		AG	6 (16.7%)	12.71	1.11	8.73%	
FL	*KCNRG*	AA	3 (6.0%)	13.50	4.33	32.07%	
		AG	22 (44.0%)	12.50	4.93	39.44%	0.00020
		GG	25 (50.0%)	11.82	5.37	45.43%	
CP	*KCNK9*	AA	20 (30.8%)	213.68	61.23	28.65%	
		AG	32 (49.2%)	246.28	82.20	33.38%	0.00113
		GG	13 (20.0%)	220.47	47.38	21.49%	
CP	*CLYBL*	CC	40 (76.9%)	207.41	62.27	30.02%	0.00010
		CT	12 (23.1%)	203.93	66.92	32.82%	

CL, cashmere length; CD, cashmere diameter; FL, fiber length; CP, cashmere production.

## 4 Discussion

NXWCG has attracted much attention because of their excellent cashmere quality. To firmly implement the country‘s agricultural policy to promote the process of agricultural and rural modernization, science and technology have been on the road. To explore the important molecular markers and candidate genes related to the fleece traits of NXWCG, the research group collected the cashmere and ear tissue samples in the early stage to analyze the phenotype traits and genotype data. By drawing the frequency distribution histogram and its fitting curve for the four phenotype traits of cashmere diameter, cashmere length, fiber length, and cashmere production, it can be seen that the phenotype data are in accordance with the normal distribution and can be used for subsequent genome-wide association analysis. A total of 288 ear tissue DNA samples shared by the four phenotype traits were selected for goat 70 K SNP chip sequencing. The results showed that 92% of the individuals were successfully genotyped, and the genotype detection rate was greater than 99%.

The basic principle of genome-wide association analysis is linkage disequilibrium. Linkage disequilibrium is a non-random association of alleles at different loci and a key indicator of population heritability (27–29). GWAS analysis does not completely rely on population pedigree information and can directly locate the target traits and SNP sites through genome-wide SNP linkage disequilibrium. GWAS analysis can also screen out the main genes that affect the complex traits of biological individuals (30, 31). The significant loci snp4438 of cashmere diameter traits is located at 38,993,710 bp on chromosome 20 of goats. This significant loci is located in the intron region of the prolactin receptor protein coding gene *PRLR*. The *PRLR* gene is the same as the candidate gene identified by the research group in Inner Mongolia Cashmere Goat. The *PRLR* gene has been confirmed to be related to the growth and development of hair follicles in cashmere goats (32). Wu et al. screened several candidate genes, such as *PRLR*, that are significantly related to hair follicle and hair growth in the study of revealing the driving factors of plateau adaptability of Jiangnan Cashmere Goat and Xizang Cashmere Goat by genome and transcriptome analysis. The finer cashmere traits of the Xizang Cashmere Goat make this breed better adapt to the cold environment of the Xizang Plateau (33).

To understand the application of candidate genes in the analysis of biological genetic mechanisms from a macro perspective, the information on key candidate genes that significantly affect the fleece traits of NXWCG was retrieved through NCBI and PubMed databases. *CCSER1* is a gene encoding a coiled-coil serine-rich protein, which is located between 33,559,344 bp and 35,024,683 bp on chromosome 6 of the goat genome. Xu et al. detected the CNV of the *CCSER1* gene in 693 individuals of 6 goat breeds by q RT-PCR and analyzed the correlation between CNV type and growth traits. The results showed that the CNV type of the *CCSER1* gene was significantly correlated with the body weight and chest circumference of GZW goats, and the expression profile showed that the *CCSER1* gene was highly expressed in the lung (34). Xue et al. used the Illumina PorcineSNP50 Bead Chip to perform genome-wide association analysis on the backfat thickness and lumbar muscle depth of 370 young eagle black pigs to detect the effects of QTL loci and candidate genes on growth traits. Finally, candidate genes related to backfat thickness, such as *CCSER1*, *GPHN*, and *MAGED1*, were screened (35). Yurchenko et al. carried out high-density genotyping and comprehensive scanning on the genomic marker selection of 15 indigenous sheep breeds in Russia. The scanning results showed that the region of the candidate gene *CCSER1* related to growth traits was consistent with the previous research results (36). *CLYBL* is a citramalyl-CoA lyase A protein, which is located between 9,645,150 bp and 9,878,794 bp on chromosome 12 of the goat genome. The gene has the molecular function of activating citrate coenzyme A activity in the gene body and the biological process involved in regulating the cobalamin metabolic process, which is involved in the regulation of protein homotrimerization and cobalamin metabolism. *CLYBL* is a mitochondrial enzyme that exists in a variety of eukaryotes and is conserved in bacteria. It is expressed in the mitochondria of various organs such as brown fat and kidney in mammals (37).

Gene Ontology describes our understanding of biology from three aspects: biological processes, cellular components, and molecular functions. Biological processes refer to biological processes completed through a variety of molecular activities, such as DNA repair or signal transduction, in a broad sense. Cell components refer to the cellular structure where gene products perform functions, such as in the endoplasmic reticulum, ribosomes, and plasma membranes. Molecular function refers to the activity of a single gene product (including proteins and RNA) or a complex of multiple gene products at the molecular level, such as “response,” “regulation,” and “catabolic process.” Gene Ontology has become one of the most popular frameworks to describe protein functions and their relationships (38–40).

The negative regulation process of cell population proliferation (GO: 0008285) enriched in this study refers to any process that can prevent or reduce the speed and degree of cell proliferation. It is a subtype of cell population proliferation regulation (GO: 0042127) and negative regulation of cell process (GO: 0048523)^3^. The genes enriched in this biological process are *ALDH1A2*, *STAT3*, *CDH13*, *NUDT6,* and *PTPN14*. Zhao et al. systematically studied the biological mechanism of hair follicle development and hair-related traits in Merino sheep based on the skin transcriptome and methylome data sets of Merino sheep and further predicted that transcription factors such as *STAT3* were involved in the morphogenesis of hair follicles during the special period of hair follicle development (41). *STAT3* has the molecular function of activating DNA binding transcription factor, RNA polymerase II specificity, and sequence-specific DNA binding in cis regulatory region of RNA polymerase II in gene ontology.

The KEGG-enriched motor protein (chx: 04814) signaling pathway (*p* < 0.05) belongs to the cell motor category in the cell process. This type of protein is involved in cell movements, such as rotational structures and structures that move along the cytoskeleton filaments. This signaling pathway was enriched in four genes: *MYO15A*, *KIF16B*, *MYO5C,* and *MYO5A*. The significant SNP loci 19: 34119983 on chromosome 19 is located in the CDS region of the *MYO15A* protein-coding gene, and *MYO15A* encodes the Myosin-XVa protein. *MYO15A* has the molecular functions of ATP binding, protein binding, and cytoskeleton movement activation in the gene ontology. The cell components include the cytoskeleton and some myosin complexes. Hadi et al. used scanning electron microscopy to measure the number and diameter of stereocilia in auditory hair cells of shake-2 mice lacking long and short Myosin-XVa protein isoforms and found that Myosin-XVa protein short isoforms are critical for controlling the size of stereocilia (42). *KIF16B* is a kinesin family member 16B protein-coding gene, and the corresponding cashmere production trait significant locus NC _ 030820.1 _ 10,060,242 is located in the intron region of this gene. *KIF16B* has the molecular functions of ATP binding, microtubule binding, phosphatidylinositol binding, and microtubule movement activation and participates in microtubule-based motor biological processes. Based on the 50 K SNP chip, Wang et al. performed a GWAS study on five wool production traits of Chinese Merino sheep, including fiber diameter, fiber diameter coefficient of variation, and fineness dispersion. The 28 significant SNPs screened at the genomic level were annotated to several candidate genes related to wool traits, such as *KIF16B* and *TCF9* (43).

## 5 Conclusion

In summary, through genome-wide association analysis of four fleece traits of cashmere length, cashmere diameter, fiber length, and cashmere production of NXWCG, a total of 18 significant SNPs were associated at the genomic level, and 232 SNPs were associated at the chromosomal level. After gene annotation of significant loci, *CLNS1A*, *CCSER1*, *RPS6KC1*, *PRLR*, *KCNRG*, *FBXO11*, *KCNK9,* and *CLYBL8* were screened as important candidate genes for fleece traits of NXWCG. Further verification of GWAS results through Sanger sequencing revealed that the mutation site of *CCSER1*, *RPS6KC1*, *KCNRG*, *KCNK9,* and *CLYBL8* genes significantly influence the fleece traits of NXWCG. The results of this study lay a theoretical foundation for further research and have extremely important scientific significance for analyzing the genetic mechanism of fleece traits.

## Data availability statement

The original contributions presented in the study are included in the article/supplementary material, further inquiries can be directed to the corresponding authors.

## Ethics statement

The animal studies were approved by Experimental Animal Management Committee of Inner Mongolia Agricultural University. The studies were conducted in accordance with the local legislation and institutional requirements. Written informed consent was obtained from the owners for the participation of their animals in this study.

## Author contributions

XL: Data curation, Formal analysis, Investigation, Methodology, Software, Validation, Visualization, Writing – original draft, Writing – review & editing. LS: Data curation, Resources, Software, Writing – review & editing. XY: Conceptualization, Formal analysis, Visualization, Writing – original draft. WL: Methodology, Software, Visualization, Writing – original draft. YS: Formal analysis, Methodology, Writing – original draft. BZ: Investigation, Software, Validation, Writing – original draft. CL: Formal analysis, Investigation, Methodology, Writing – original draft. LY: Formal analysis, Methodology, Writing – original draft. JW: Formal analysis, Investigation, Methodology, Writing – original draft. DJ: Investigation, Writing – original draft. RC: Investigation, Writing – original draft. AC: Investigation, Writing – original draft. BG: Data curation, Investigation, Writing – original draft. ZW: Methodology, Validation, Visualization, Writing – original draft. WJ: Formal analysis, Writing – original draft, Writing – review & editing. YW: Project administration, Resources, Writing – original draft, Writing – review & editing. RS: Funding acquisition, Methodology, Project administration, Writing – review & editing, Writing – original draft.

## Footnotes

^1^ http://www.ncbi.nlm.nih.gov

^2^ http://david.abcc.ncifcrf.gov

^3^ https://www.ebi.ac.uk/QuickGO
